# Does the transfer of knowledge from the pioneer generation to the second‐generation speed‐up the learning curve of robot‐assisted partial nephrectomies? TRANSFER trial (UroCCR n°83)

**DOI:** 10.1002/bco2.477

**Published:** 2024-12-11

**Authors:** Louis Vignot, Zine‐Eddine Khene, Adil Mellouki, Arnoult Morrone, Jean‐Christophe Bernhard, Karim Bensalah, Daniel Chevallier, Nicolas Doumerc, Morgan Roupret, Francois‐Xavier Nouhaud, Cédric Lebacle, Jean‐Alexandre Long, Pierre Pillot, Xavier Tillou, Brannwel Tibi, Matthieu Durand, Younes Ahallal, Imad Bentellis

**Affiliations:** ^1^ Service d'Urologie, Andrologie, Transplantation Rénale, Hôpital Pasteur 2, CHU de Nice Nice France; ^2^ Department of Urology University Hospital of Rennes Rennes France; ^3^ Department of Urology University Hospital of Bordeaux Bordeaux France; ^4^ Department of Urology University Hospital of Toulouse Toulouse France; ^5^ Department of Urology University Hospital of Paris Paris France; ^6^ Department of Urology University Hospital of Rouen Rouen France; ^7^ Department of Urology University Hospital of Grenoble Grenoble France; ^8^ Department of Urology University Hospital of Poitiers Poitiers France; ^9^ Department of Urology University Hospital of Caen Caen France; ^10^ INSERM U1081 ‐ CNRS UMR 7284 Université Cote d'Azur Nice France

**Keywords:** learning curve, partial nephrectomy, pedagogy, robotics

## Abstract

**Objectives:**

The objective is to compare the learning curves between two pioneer and three second‐generation surgeons for RAPN in terms of WIT, CD and positive surgical margins.

**Materials and methods:**

The charts of consecutive RAPNs of three centres were reviewed from the UroCCR prospective database. The experience was assessed by a regression model for each group. There was a univariate analysis on three consecutive sequences of 15 procedures. The learning speed for WIT was explored graphically by polynomial regression after cubic splines. Finally, CUSUM charts were obtained.

**Results:**

There were 1203 RAPN in the pioneer group and 119 performed by second‐generation surgeons. There was a significant difference in the distribution of tumour size (*p* < 0.001) and the RENAL score (*p* < 0.001). The operative time was longer in the first group (*p* > 0.001). Independent factors for a higher WIT were the second group (*p* < 0.001), higher experience (*p* < 0.001) the collinearity between the group and experience (*p* < 0.001), the RENAL score (*p* < 0.001) and blood loss (*p* < 0.001). Adjusted Loess regressions showed a plateau of WIT at 400 procedures for the pioneers and a significant decrease at 20 procedures for the second generation. CUSUM chart analysis showed a ‘staircase’ pattern of the learning process, with three major steps at 150, 200 and 300 procedures. The major limitation is the difference in sample size between the two arms.

**Conclusions:**

Learning curve patterns would reflect a transfer of knowledge to the second‐generation, as opposed to the establishment of standards by the pioneers.

## 1 INTRODUCTION

The number of procedures a surgeon may have to carry out before reaching a safe, and competent level of performance is fundamental to aid in clinical decision‐making and healthcare policy. This phenomenon is termed the surgical learning curve (LC). Partial nephrectomy (PN) is the standard treatment of localised renal tumours. During the last decade, robotic assistance has increasingly been adopted. Nonetheless, this procedure is subjected to the surgeon's expertise and skills, and the LC has been pointed out as a limitation to the diffusion of this surgical technique.^1^, ^2^, ^3^ Recently, Larcher et al.^4^ highlighted that surgeon experience (EXP) was associated with a shorter warm ischemia time (WIT) and a lower probability of Clavien‐Dindo (CD) complications ≥2, but not with a reduction in positive surgical margins (PSM) rates. Also, the relationship between EXP and WIT appeared to be nonlinear, with a plateau observed after 150 procedures, whereas the relationship between EXP and CD ≥2 was linear, without reaching a plateau at 300 procedures. All these elements allow us to question the trifecta (WIT, CD, PSM) as a composite factor and explore each of the components separately with orthogonal statistical methods.

We also wanted to address the question of hospital volume. We assume that, in medium‐volume centres, a second generation of surgeons, benefiting from the experience of the pioneers, could achieve equally satisfactory results. This is especially true since initial training with an ERUS‐type curriculum^5^ would reduce the risk of suboptimal results due to the initial LC.

The main objective of this study is to compare the LCs between two pioneer surgeons and three second‐generation surgeons for RAPN in terms of WIT, CD and PSM according to several statistical approaches.

## 2 PATIENTS AND METHODS

### 2.1 Study design

All patients in this study were prospectively enrolled in the UroCCR multicentric database (ClinicalTrials.gov: NCT03293563/CNIL agreement DR‐2013‐206). We conducted a retrospective analysis of patients who underwent RAPN for a localised kidney tumour at three academic departments of urology between 2012 and 2021. Patients who had multiple masses were excluded from the analysis to minimise the confounding effect on perioperative outcomes. All RAPN procedures were performed using the da Vinci surgical system through a transperitoneal approach. Two pioneers in kidney cancer robotic surgery, each from a different centre, were chosen for their experience in the field of robotic surgery and their involvement in renal oncology. The three second‐generation surgeons were all from the same university centre. They had previous experience of robotic surgery and laparoscopic PN. The beginning of the LC corresponded to the implementation of robotic activity in the centre in 2017.^6^

### 2.2 Covariables

The following preoperative data were collected prospectively for each patient in the UroCCR database: age; sex; weight; anticoagulant/antiplatelet intake; American Society of Anesthesiology (ASA) score^7^; TNM classification; Tumour size; RENAL score. The following surgical data were recorded: operative time, on or off‐clamp procedure, WIT, blood loss and transfusion. The surgical experience was determined for each surgeon by counting from the first procedure recorded in UroCCR. We carefully included surgeons for whom this information was available and who are well‐known to be pioneers (JCB, KB) and second generation (MD, BT and YA).

Postoperative data PSM, length of stay, complications within 30 days, creatinine and haemoglobin levels were assessed. Postoperative complications were graded using the CD classification.

### 2.3 Outcomes of interest

The primary endpoints were the elements of the trifecta assessed separately.^8^ Warm ischemia time (WIT), post operative complications graded according to the Clavien‐Dindo classification.^9^ Finally, PSM were obtained from the pathology records and prospectively fed into the database.

### 2.4 Statistical analysis

Means and standard deviations were reported for continuous variables, and frequencies and proportions for nominal variables. Fisher's exact test was used to compare nominal variables, the Kruskal–Wallis test for variables with more than two strata and Student's *t*‐test to compare quantitative continuous variables. Non‐parametric tests were performed for data with small sample sizes (<30).

The analysis was designed in five steps. The first one was a comparison of the two groups including all patients (Table 1). The same comparison was performed by including only the first 120 patients (KB 60 + JCB 60) of the pioneers to explore comparability of the same starting experience.

**TABLE 1:** Comparison between generations.

		2nd generation (ref group)	Pioneers (overall)	*p*	Pioneers (first experience)	*p*
		119	1203		120	
Preoperative characteristics						
Age (mean‐sd)		63.5 (±10.4)	59 (±13.3)	**<0.001**	61 (±14)	**0.019**
Sex (%)		51 (42.9)	454 (37.7)	0.319	49 (40.8)	0.852
Weight (mean‐sd)		77.6 (17.8)	81 (18.4)	0.082	78 (17.5)	0.855
ASA (%)	1	4 (4.5)	226 (19.1)	**<0.001**	26 (22.8)	**<0.001**
	2	43 (48.9)	740 (62.4)		56 (49.1)	
	3	41 (46.6)	215 (18.1)		31 (27.2)	
	4	0 (0.0)	4 (0.3)		1 (0.9)	
TNM (T) (%)	T1a	44 (41.5)	596 (50.7)	**<0.001**	63 (57.8)	**<0.001**
	T1b	36 (34)	426 (36.2)		43 (39.4)	
	T2a	9 (8.5)	90 (7.7)		3 (2.8)	
	T2b	1 (0.9)	28 (2.4)		0 (0.0)	
RENAL Score (mean‐sd)		5.7 (2.6)	7.4 (2.2)	**<0.001**	6.70 (2.9)	**0.03**

Operative outcomes						
Operative time—min (mean‐sd)		150.3 (107.2)	193.1 (89.6)	**<0.001**	218.9 (103.6)	**<0.001**
On‐clamp (%)	yes	100 (84)	759 (63.4)	**<0.001**	118 (99.2)	**<0.001**
WIT—min (mean‐sd)		20.81 (9)	22.60 (11)	0.124	18.54 (8)	**0.05**
Blood loss—mL (mean‐sd)		308.02 (313)	347.46 (377)	0.285	377.25 (461)	0.187
Transfusion (%)		5 (4.3)	37 (3.1)	0.673	8 (6.7)	0.601

Follow‐up						
Hospital stay (median‐IQR)		2 [2, 3]	2 [1, 4]	0.078	5 [4, 6]	**<0.001**
Clavien (%)	1	1 (50.0)	23 (32.9)	0.687	5 (38.5)	0.326
	2	0 (0.0)	24 (34.3)		5 (38.5)	
	3	1 (50)	6 (8.6)		1 (7.7)	
	3a	0 (0.0)	5 (7.1)		2 (15.4)	
	3b	0 (0.0)	6 (8.6)		0 (0.0)	
	4a	0 (0.0)	3 (4.3)		0 (0.0)	
	4b	0 (0.0)	1 (1.4)		0 (0.0)	
	5	0 (0.0)	2 (2.9)		0 (0.0)	
Creatinin μmol/L Day1 (mean‐sd)		92.83 (24.6)	110.34 (54)	**0.003**	109.28 (45)	**0.002**
Haemoglobin g/dL Day1 (mean‐sd)		12.59 (1.6)	12.62 (1.6)	0.902	12.21 (1.69)	0.111
PSM (%)		14 (11.8)	53 (4.4)	**0.001**	4 (3.3)	**0.03**
Focal recurrence (%)		0 (0.0)	187 (17.3)	**<0.001**	17 (16)	**<0.001**
Metastatic progression (%)		0 (0.0)	161 (14.9)	**0.001**	11 (10.4)	**0.01**
Overall survival (%)		0 (0.0)	40 (3.6)	0.180	14 (11.8)	**0.005**
Follow‐up months (median‐IQR)		13 [12, 23]	17 [6, 35]	0.62	37 [26, 58]	**<0.001**

Abbreviations: PSM, positive surgical margin; WIT, warm ischemia time.

Multivariable analysis was performed to assess the impact of EXP on achieving each element of the trifecta. Logistic regression was performed to seek predictors of PSM and CD. A general linear model was used to seek predictors of WIT. Only clinically relevant variables or with *p*‐value <0.25 in univariate analysis was included in the multivariate model. Collinearities were included in the initial model, and the factors known from the existing literature (RENAL score, age, EXP) were constrained in the final model.

Thirdly, we refined the comparison between the groups according to three consecutive sequences of 20 procedures of experience to assess the progressive differences.

We plotted the LC of estimated WIT adjusted on RENAL and age using polynomial regression after cubic splines. This same method from Larcher et al.^4^ allowed us to plot the curve of the WIT, by cancelling the specific effect of confounding factors. Off‐clamp procedures were not removed from this analysis.

Finally, we performed a CUSUM analysis^10^ for the three outcomes to look for changes in the learning process. This is a robust method to determine deviations from a mean in time series analysis. Non‐risk‐adjusted CUSUM methods were used. For binary outcomes, the cumulative CUSUM curve was generated. Control limits for binary outcomes were set to be five standard errors (SE) of the summary statistics. For continuous outcomes, the standard CUSUM curve was also generated as well as the xbar charts, which plots the variable over time. Control limits for continuous outcomes were also set to detect outcome values five SE from the target process mean.

Statistical analyses were performed using R statistical software.^11^ All tests were two‐sided with a significance level at *p* < 0.05.

## 3 RESULTS

### 3.1 Patient characteristics

**FIGURE 1:**
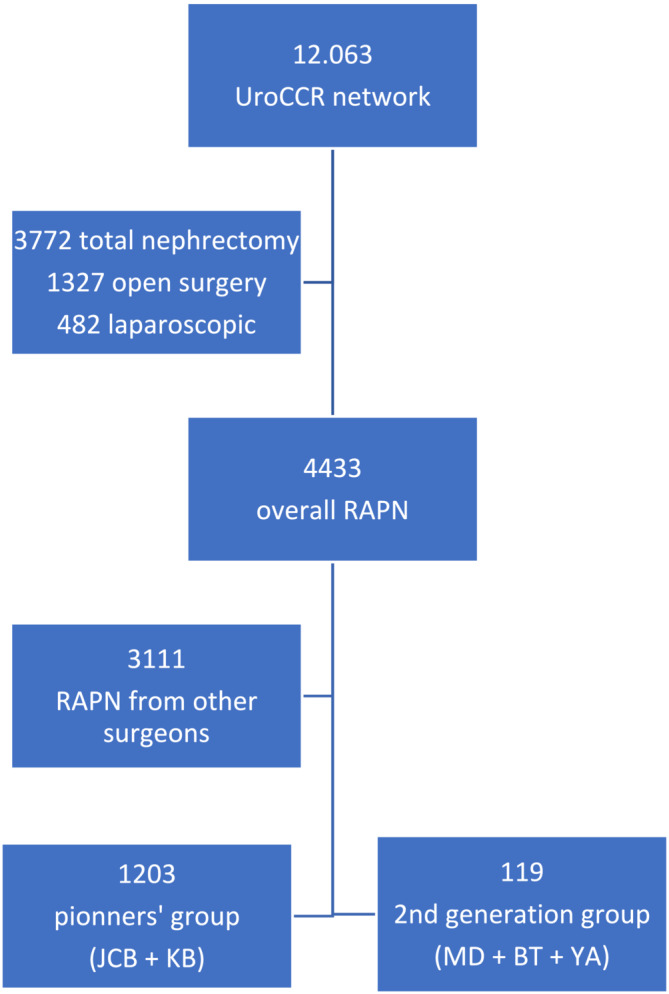
Flow chart. RAPN, robot‐assisted partial nephrectomy.

Out of 1322 RAPN, 1203 in the pioneering group and 119 performed by second‐generation surgeons (Figure 1), there was a significant difference in the distribution of tumour size (first: 3.95 vs. second: 7.82 cm, *p* < 0.001) and the RENAL Score (first: 5.7 vs. second: 7.4, *p* < 0.001). The operative time was longer in the first group (193 vs. 150 min, *p* < 0.001). The main renal artery clamping was the principal type of hilar control approach in both groups (first: 63% vs. second: 84%, *p* < 0.001).

PSM rate was lower in first group (4.4% vs. 11.8%, *p* = 0.001), whereas no statistically significant differences were found for WIT, hospital stay and CD (*p* = 0.1, 0.07 and 0.6, respectively) (Table 1).

When considering only the first 120 patients of the pioneer group (first), thus representing the beginning of their LC, the RENAL score was still higher (first: 6.7 vs. second: 5.7 *p* = 0.03), but the rate of on‐clamp procedures is inverted in favour of first group (99.2% vs. 84% *p* < 0.001). Operative time and hospital stay were longer for the pioneer surgeons (*p* = 0.001). The PSM rate was consistently lower in the pioneer group, while the WIT and CD rates remained comparable (Table 1).

### 3.2 Multivariate assessment of the experience

Independent factors for a higher WIT were the second group (*p* < 0.001), greater experience (*p* < 0.001), the collinearity between the group and experience (*p* < 0.001), the RENAL score (*p* < 0.001) and blood loss (*p* < 0.001). However, when experience was removed from the model, the group variable was no longer significantly associated with WIT.

For CD, the model did not converge with the group variable, due to few CD in Group 2. However, the only associated factor was blood loss (*p* = 0.03), as well as for PSM (*p* = 0.005).

### 3.3 Sequential assessment of experience

We refined the univariate comparison between the groups, considering three blocks of 20 consecutive procedures, thus defining three experience steps (Table 2).

**TABLE 2:** Sequential comparison of generations.

		Second generation	Pioneers	*p*
First sequence of experience 1 to 20				
RENAL Score (median‐IQR)		6 [4, 6.25]	6 [0, 8]	0.50
Operative time—min (median‐IQR)		155 [120, 200]	195 [165, 270]	**0.003**
Blood loss—mL (median‐IQR)		225 [100, 425]	175 [100, 425]	0.627
WIT—min (median‐IQR)		22 [17.75, 28.25]	18 [14.5, 24]	**0.032**
PSM (%)		9 (15)	3 (7.5)	0.414
Clavien (%)	1	1 (50)	0 (0.0)	0.233
	3	1 (50)	1 (33.3)	
	3b	0 (0.0)	2 (66.7)	

Second sequence of experience 21 to 40				
RENAL Score (median‐IQR)		5 [5, 7]	8 [6, 10]	**0.004**
Operative time—min (median‐IQR)		120 [90, 150]	183 [142.5, 335]	**<0.001**
Blood loss—mL (median‐IQR)		120 [90, 150]	183 [142.5, 335]	**<0.001**
WIT—min (median‐IQR)		15 [11, 22]	18 [14, 19.5]	0.738
PSM (%)		4 (10.3)	1 (2.5)	0.340
Clavien (%)	2	0 (NaN)	3 (100)	NaN

ThIrd sequence of experience 41 to 60				
RENAL score (median‐IQR)		8 [6.5, 8]	7 [6, 8]	0.675
Operative time—min (median‐IQR)		105 [87.5, 150.25]	182.5 [120, 299.75]	**0.001**
Blood loss—mL (median‐IQR)		200 [137.5, 462.5]	200 [200, 300]	0.542
WIT—min (median‐IQR)		13 [10.5, 20.75]	18 [11, 20.5]	0.220
PSM (%)		1 (5)	0 (0.0)	0.721
Clavien (%)	2	0 (NaN)	2 (28.6)	NaN

Abbreviations: PSM, positive surgical margin; WIT, warm ischemia time.

In the first sequence, RENAL scores were similar (first: 6 vs. second: 6, *p* = 0.5), the operative time was shorter in the second group (195 vs. 155 min, *p* = 0.03), the WIT was shorter for pioneers (18 vs. 22 min, *p* = 0.03), while the PSM and CD rates were comparable.

In the second sequence, the pioneer surgeons shifted for more complex tumours (RENAL score 8 vs. 5, *p* = 0.004), but the WIT remained equivalent (first: 18 vs. second: 15, *p* = 0.74), while keeping the same observation as the first sequence concerning operative time, PSM and CD rates.

In the third sequence, second‐generation surgeons were the same as the pioneers for the complexity of the tumours (RENAL score first: 7 vs. second: 8, *p* = 0.6), with comparable results in in terms of WIT, PSM and CD rates, while keeping a significant difference in terms of operative time (first: 183 min vs. second: 105 min, *p* = 0.001).

### 3.4 LC for WIT

The plot of WIT according to experience after adjusting for RENAL score and age showed a plateau shape at 25 min after 400 procedures for the pioneers, while it showed a consistent decrease after 20 procedures for the second generation (Figure 3).

### 3.5 CUSUM analysis

**FIGURE 2:**
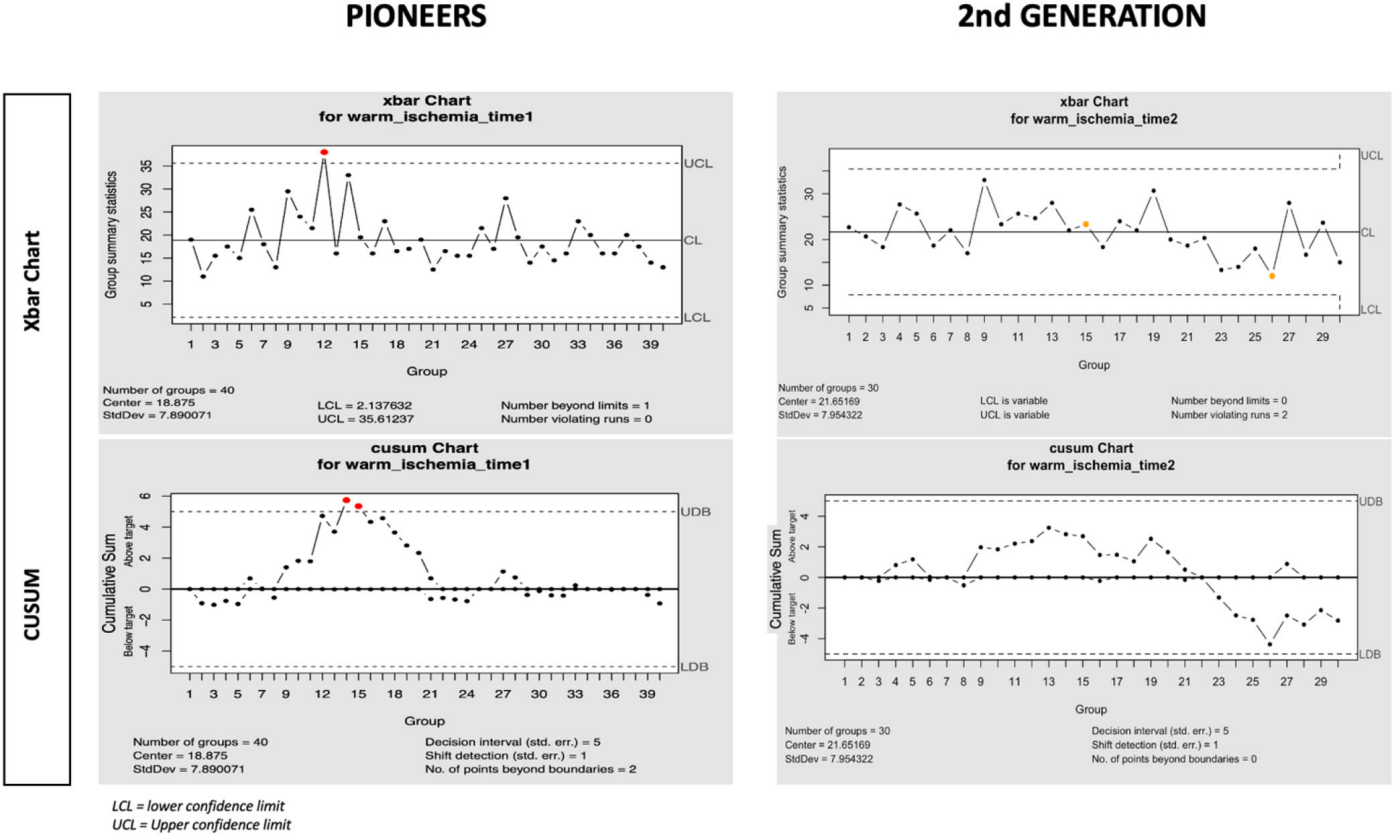
Xbar chart and Cusum chart of warm ischemia time according to the first 30 procedures.

The patterns of the CUSUM charts of WIT for the first 30 procedures are different between the groups. For the pioneers, the process was stable around an average time of 20 min, with an outlier at the twelfth procedure. However, for the second generation, we observed a persistent decrease in WIT after 20 procedures (Figure 2).

The charts for CD and PSM are harder to interpret because of the small number of events. It shows however a decrease after 20 procedures for the second generation as well as for the pioneers.

Finally, the analysis of the overall experience of the pioneers for each of the trifecta parameters exhibited a ‘staircase’ pattern. The learning process seemed evolve through major steps. We observed three spikes of a worsening in the results (approximately at 150, 200 and 300 procedures) after three corresponding sequences of improvement.

## 4 DISCUSSION

**FIGURE 3:**
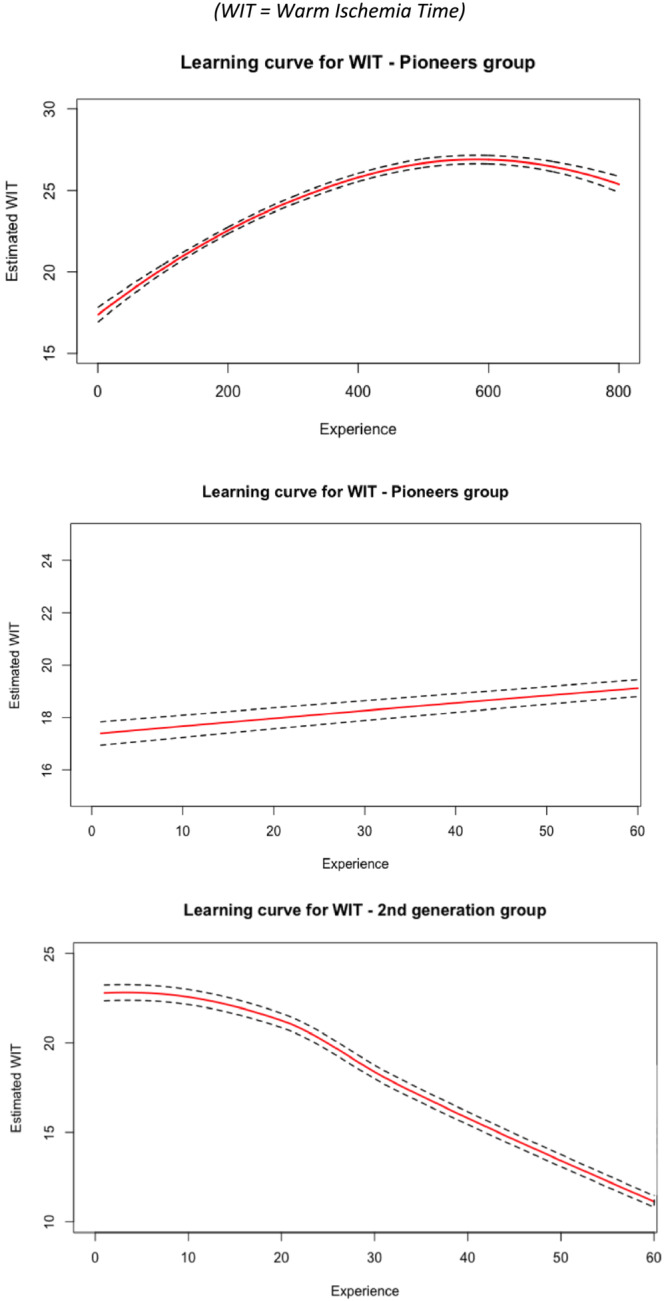
Polynomial regression after cubic splines. (WIT, warm ischemia time).

Our findings support a nonlinear and non‐homogenous relationship between EXP and WIT. For the second generation (Group 2), there was a steep improvement in WIT after 20 procedures. This finding is consistent across different methods (Table 2, Figure 2). The sequential model (Table 2) shows a relatively better improvement of WIT across the three sequences of 20 procedures. This is probably due to the earliest use of the off‐clamp technique. Concerning the pioneers (Group 1), Figure 3 surprisingly shows an increase in WIT over the first 400 procedures to reach a 25 min plateau. Even when this regression was adjusted on RENAL score and age, it did not completely account for the overall surgical complexity. Indeed, the CUSUM charts exhibit a staircase pattern, suggesting new challenges throughout the learning process. The multivariate analysis supports this claim by showing that greater experience is counterintuitively associated with longer WIT. This is particularly true for the pioneers, who had lower WIT than the second generation at the beginning of the LC. It also explains why the second generation are associated with longer WIT (OR 9.7). When EXP was removed from the model, the group variable was no longer significantly associated with WIT. This suggests that the inertia of the EXP variable is higher in the first group because of the asymmetry in sample size between arms and the robustness of the estimation. This means that Pioneers have an overall better WIT estimated on a longer period of time, but the second generation shows faster improvement over the first procedures.

No definitive conclusion could be made for CD because of the lack of events, in particular for the second group. For the pioneers, the LC suggests constant improvement as pointed out by Larcher et al. We did not present here the curves for CD. However, the CUSUM chart (Figure 3) showed this improvement. It also adds to the staircase pattern outcome.

PSM rates were higher for the second generation (11.8% vs. 4.4%), with an improvement over the first three sequences of 20 procedures (Table 2), respectively, at 15%, 10% and 5%. Pioneers are consistent from the starting of the LC. These findings suggest an attempt for the second generation to rapidly achieve the standards set by the pioneers, in particular the challenges that the pioneers took longer to face (complex masses, enucleation, off‐clamp technique^12^).

These outcomes raise the question about the interpretation of the trifecta (WIT, CD, PSM) and the complexity of its composite value as pointed out by Jens et al.^13^, ^14^ or Cadeddu et al.^15^ They agreed that WIT should no longer be a primary element in learning objectives, given its weak involvement in long‐term nephron saving^4^ compared to other factors.^16^, ^17^, ^18^ On the other hand, it is easy to imagine that increased experience is associated with greater challenges. Indeed, Paulucci et al.^2^ have shown a change in tumour and patient characteristics after the initial learning phase, with larger tumours and more fragile patients.

The second issue concerns the statistical approach introduced by Vickers et al.^19^ The interpretation of the plateau shapes is also linked to this type of analysis. Comparison with other statistical tools should strengthen the robustness of this result and diversify its discussion.

The differences due to asymmetry between arms led to the question of the volume of activity. Indeed, the learning process is not supported by the surgeon alone. The team also develops expertise in the robotic surgery and the interaction with the surgeon's methods. The outcomes here illustrate this difference between the two groups. The practical problem of small to medium‐volume centres concerns essentially second‐generation surgeons and/or the development of new activities. Recently, Motoyama et al.^20^ analysed the first 65 consecutive procedures of an experienced PN surgeon. A console time ≤150 min and WIT ≤20 min was obtained at the sixth and fourth procedures, respectively. For Omidele et al.,^8^ the learning plateau was reached between 61 and 90 procedures after 66 to 80 months based on a volume of 20 cases per year. A study^21^ of 43 178 partial nephrectomies including 2187 RAPN between 2008 and 2011 showed that the median annual hospital volume was 27 cases (interquartile range: 11–64). There was a nonlinear association between annual volume and the complication rate. The plateau corresponded to 18 to 20 cases per year. Moreover, caseload is a dynamic process that evolves in parallel with surgeon EXP. In our centre, at the beginning of the activity, the caseload was 10 cases per year, and now, it is 65 cases per year.^6^

Our study has several limitations that should be acknowledged. First, we excluded multiple and benign tumours, and the retroperitoneal approach in order to work on a more homogenous series of RAPN patients. This limits the generalisation of the results,^22^ but it gives better control over their interpretation. In the same way, we missed data about toxic fat to assess the difficulty^23^ or the enucleation technique.^24^ Secondly, we believe that a major drawback remains the asymmetry in arm size. We assume that follow‐up is not highly important regarding the immediate outcomes of the trifecta, but the sample size difference complicates the interpretation of the results and the relative impact of confounders. We believe that multiple orthogonal methods provided more precise insight into the structure of the data and enhanced the discussion. Thirdly, EXP is a fuzzier concept than one might think. Information about previous experience in laparoscopic or other robot‐assisted surgery modalities was missing. Dias et al.^25^ showed that surgeons trained in LPN obtained satisfactory results at about 44 RAPN. Also, information about the involvement of a second surgeon or a trainee was not available. One could argue that proctoring a trainee leads to worse results, but proctoring is also a proxy for experience. We could not state if it is an authentic bias, nor its direction. Finally, the LC of the team is important, and we suppose that it grows alongside with the surgeon's LC. It is also linked to the annual caseload of the centre. These factors could explain the non‐homogenous trend in LC as well as the modification of the technique and the material used over time.

This work compared the initial LC of a second generation of surgeons with pioneers, but we are now watching a third‐generation learning. The use of the simulator and proctoring resources is extensive. These educational programmes are part of the development of a multi‐surgery robotic platform and the optimisation of team interactions.

## 5 CONCLUSION

We have shown that second‐generation surgeons are faster at implementing the tools developed by a pioneer generation. When pioneers showed consistent results over time with specific steps of increasing challenges, the second generation faces these challenges earlier with worse results at the beginning, but very fast learning over 20 procedures. Training and proctoring programmes could supervise this initial period. LC is a complex cognitive phenomenon involving multiple parameters of difficulty, nonlinear knowledge acquisition and interaction with a team. The trifecta is an overall outcome of performance but does not fully reflect the challenges of RAPN surgery.

## AUTHOR CONTRIBUTIONS

Louis Vignot has full access to all the data in the study and takes responsibility for the integrity of the data and the accuracy of the data analysis.

*Study conception and design*: L. Vignot, I. Bentellis, Z. Khene, D. Chevallier, JC Bernhard, K. Bensalah, B. Tibi, M. Durand and Y. Ahallal. *Drafting of the manuscript*: I. Bentellis, Y. Ahallal and M. Durand. *Critical revision of the manuscript for important intellectual content*: Z. Khene, A. Mellouki, A. Morrone, D. Chevallier, JC Bernhard, K. Bensalah, B. Tibi, M. Durand, Y. Y. Ahallal, N. Doumerc, M. Roupret, F.X. Nouhaud, C. Lebacle, J.A. Long, P. Pillot and X. Tillou. *Statistical analysis*: I. Bentellis and Z. Khene. *Obtaining funding*: None. *Administrative, technical or material support*: None. *Supervision*: Y. Ahallal and I. Bentellis. *Other*: None.

## CONFLICTS OF INTEREST STATEMENT

Louis Vignot certifies that all conflicts of interest, including specific financial interests and relationships and affiliations relevant to the subject matter or materials discussed in the manuscript (e.g., employment/affiliation, grants or funding, consultancies, honoraria, stock ownership or options, expert testimony, royalties, or patents filed, received, or pending), are the following: Branwell Tibi/span> and Matthieu Durand discloses proctoring activity for Intuitive Surgical. Karim Bensalah discloses consulting activity for Intuitive Surgical.
